# Properties, Applications and Toxicities of Organotrialkoxysilane-Derived Functional Metal Nanoparticles and Their Multimetallic Analogues

**DOI:** 10.3390/ma16052052

**Published:** 2023-03-02

**Authors:** Prem C. Pandey

**Affiliations:** Department of Chemistry, Indian Institute of Technology (BHU), Varanasi 221005, India; pcpandey.apc@iitbhu.ac.in

## 1. Introduction

Sol–gel chemistry offers a very unique tool for nanoscale mastering of material preparation from metal alkoxides. However, when metal is replaced by silica, the resulting process under acid/base catalysis allows the formation of nanostructured silicate with exceptional properties, and the same process is followed in natural material evolution. As a consequence, important functionalities meeting the requirement of Lewis acid/Lewis base character in alkoxide science have been innovated via the introduction of reactive organotrialkoxysilanes to derive nanostructured silicates with controlled morphology [1,2,3]. The presence of suitable functionalities not only controls the sol–gel processing of trialkoxysilane but also enables specific interaction, leading to the introduction of a porous morphology that meets the in situ requirement of innovative technological applications based on immobilization technology. There are four types of such functionalities: 3-aminopropyltrialkoxysilane, [2-(3,4-Epoxycyclohexyl)ethyl]trimethoxysilane, 3-Glycidoxypropyltrimathoxysilane and trimethoxysilane are primarily discussed and explored in our group as reactive organic functionalities for yielding nanostructured domains in a variety of formulation.

## 2. Functional Organotrialkoxysilane-Derived Biocompatible Organically Modified Silicate Thin Films

The role of organically modified silicate films (ORMOSILs) derived from the selective interaction of specific organic functionalities linked to trialkoxysilane [3,4,5,6,7,8,9,10] exploring the concept of wettability has been precisely explored in our group [3]. 3-Aminopropyltrimethoxysilane and [2-(3,4-Epoxycyclohexyl)ethyl]trimethoxysilane were found to undergo specific interaction based on their Lewis acid or Lewis base character, forming polymeric domains of an in situ-generated morphology as a function of targeted sensing components, followed by the introduction of -Si-O-Si- domains of a controlled nanostructured network of organically modified silicates under ambient conditions, while retaining the biological activity of the sensing components [3]. This provides a novel approach for integrating sensing components together with physio-chemical transducers, justifying its use in a variety of applications in the following areas: (1) nanostructured domains based on organically modified silicates for chemical sensing; (2) encapsulation/immobilization of sensing components within organically modified silicates; and (3) manipulation of nanostructured networks of organically modified silicate films via the incorporation of suitable reagents, which has been innovated and developed at Banaras Hindu University, revealing a very convenient way for deriving nanomaterials for practical applications, especially in the fabrication of chemical sensors. This reflects the theme of this Special Issue on the future design of nanomaterials.

## 3. Reactive Organotrialkoxysilane-Derived Metal Organic Framework in Nanostructured Network of Organically Modified Silicate

Electrochemical sensors based on mediated electrochemistry has been extensively studied; they involve the participation of an organic compound, such as ferrocene, derivatives of ferrocene, TCNQ and TTF, together with a biological sensing component within a immobilized membrane matrix to eliminate the limitations of first-generation enzyme electrodes [3]. When such configuration was tried in an organically modified silicate matrix, electrochemical behaviour was normally associated with the sluggish redox electrochemistry of encapsulated redox molecules [3]. On the other hand, these materials had already been proven to be an efficient combination, displaying excellent redox bioelectrochemistry in the introduction of a commercial blood glucometer for sensing blood glucose level in a drop of blood sample (video link: https://youtu.be/rVydRLrGOY8, accessed on 1 January 2023). The sluggish electrochemistry was found to be associated with the availability of poor electron hopping sites within the ceramic matrix, which was subsequently innovated via the introduction of electrocatalysts, such as palladium. We innovated the specific interaction between palladium cations with the reactive organic precursors of ormosil, such as 3-glycidoxypropyltrimehoxysilane and [2-(3,4-Epoxycyclohexyl)ethyl]trimethoxysilane, leading to the reduction of these cations into palladium nanoparticles [3]. These reactive moieties allowed the formation of palladium nanoparticles, which subsequently enabled the formation of –Pd-C- linkage, generating palladium-linked ormosil [3]. This finding was not limited to such innovative chemistry; while dealing with reactive trialkoxysilane as one of the hydrophilic components in ormosil formation, the introduction of -Pd-Si- linkage, along with -Pd-C- linkage, within the nanostructured network of ormosil converted the nanostructured silicate into a solid solution that displayed excellent redox electrochemistry of ferrocene monocarboxylic acid; this redox electrochemistry was even better than that recorded in the homogeneous solution when another hydrophilic ormosil precursor, trimethoxysilane, specifically reacted with palladium chloride to form -Pd-Si- bonding during the sol–gel processing of ormosil. These findings allowed the design of a library of electrocatalytic sites within nanostructured domains for a variety of applications and justified their significance in a many applications [3].

## 4. Reactive Organotrialkoxysilane-Mediated Synthesis of Processable Monometallic, Bimetallic and Trimetallic Noble Metal Nanoparticles

The conventional ways of synthesizing metal nanoparticles have recently been reviewed [3] for their use as early as from the 4th century to the 21st century [5,6,7,8,9,10], with the following limitations: (i) Generally, AuNPs are produced as an aqueous suspension, and the use of such NPs in an organic solvent causes agglomeration; similarly, NPs having compatibility with organic solvents (Brust–Schiffrin method) are not compatible in an aqueous system. (ii) NPs fabricated through conventional routes are generally produced as a dilute solution; the initial concentration of these NP precursors is very low. (iii) NPs fabricated through conventional routes are not very stable to changes in pH- and salt concentration, which limits their use in several applications as these NPs have to be modified to serve the purpose; for example, NPs made through Turkevisch method agglomerate upon the addition of single drop of salt. NPs should be able to adapt to different conditions (change in solvent, change in pH, and change in salt concentration) without undergoing any change in their size or shape in real applications. (iv) Attempts to convert the homogenous suspension of NPs to heterogeneous matrix by adsorbing over some solid support (TiO_2_, Al_2_O_3_, etc.) cause an increase in the size of these NPs, i.e., they undergo agglomeration. To meet these challenges, sol–gel science and technology, which is one of the most widely accepted methods for creating nanomaterials, seems to be reasonable to use, as evident from the variety of functional activities available with the use of reactive trialkoxysilane as a potent reducing agent for palladium cation [3]. Accordingly, such findings point to the importance of examining the role of functional trialkoxysilane during the synthesis of noble metal nanoparticles; indeed, the real-time synthesis of noble metal nanoparticles, together with their multimetallic analogues, has been reported, involving the active role 3-APTMS and 3-GPTMS for the first time under ambient conditions [3]. Further innovation of noble metal nanoparticle synthesis incorporated microwave exposure of 3-APTMS and 3-GPTMS modulated nanoparticle synthesis within a few seconds [3] [YouTube link: https://youtu.be/Zl-QT574j8Q, accessed on 1 January 2023]. The findings reported in [3] described the use of two functional alkoxysilanes as the reducing and stabilizing agents, overcoming the limitations of conventional routes of synthesizing noble metal nanoparticles and their multimetallic analogues. These findings precisely demonstrated the role of functional alkoxysilanes in assisting in the controlled and rapid synthesis of noble metal nanoparticles and their multimetallic analogues. Although the use of functional trialkoxysilanes to yield Ag-NPs or other noble metal nanoparticles are most effective; however, the Ag-NP formulation that can be converted into (i) thin film over the surface for biomedical applications, such as surgical catheter or normal catheter; (ii) Ag-NP spray, allowing the assembling of Ag-NPs over any desired substrate just by spraying; and (iii) embedment of Ag-NPs over nylon cloth or other cotton fabrics. These are some of the further potential applications of organotrialkoxysilane-derived nanomaterials [YouTube link: https://youtu.be/ViQ9ivQ8msg, accessed on 1 January 2023].

## 5. Reactive Organotrialkoxysilane-Derived Self-Assembling Siloxane–Nanoparticle Nanofluid

Recently, acetone-induced polymerization of 3-APTMS in chloroform has been observed, leading to the formation of siloxane. Using ^1^H, ^13^C and ^29^SiNMR techniques, it has been proven that acetone reacts with the amino group of alkoxysilane to form an imine [(CH_3_)_2_C=N(CH_2_)_3_Si(OCH_3_)_3_], IPTMS or N-isopropylidene-3-aminopropyltrimethoxysilane. The water that is released during the imine formation hydrolyzes methoxysilane, thereby inducing the formation of siloxane and Si-O-Si bridges. Furthermore, we have also observed that IPTMS formed from 3-APTMS and acetone shows catalytic activity by reducing noble metal cations into their respective nanoparticles during the synthesis of AuNPs, which might be useful in simultaneously forming AuNP–siloxane nanofluid, given the bifunctional nature of 3-APTMS. Corroborating with these findings, the synthesis of spherical AuNPs utilizing the interaction between 3-APTMS and acetone has been demonstrated [3]. These findings inform our understanding of gold cation-induced polymerization of indole monomer, which forms a Lewis acid–base adduct with gold cations to form a polyindole–gold nanoparticle suspension. This approach may subsequently enable the specific interaction of two colloidal suspensions in acetone, siloxane–gold nanoparticles and the polyindole–gold nanoparticle sol, to yield a polymeric nanofluid of siloxane–polyindole–gold nanoparticles. This type of conversion has been recorded in other volatile solvents, such as chloroform, acetonitrile and dichloromethane. The polyindole–gold nanoparticle suspension undergoes self-assembly with the siloxane–gold nanoparticle suspension, yielding a polymer nanofluid that is suitable for making membranes and coatings; the properties of these materials may be tuned with a suitable organic molecule that exhibits the desired functionality. Such materials open a new horizon for developing extremely stable membranes for biosensor design that are analogous to biological membranes in thickness and also for realizing the analogous interaction of channel-forming proteins.

## 6. Synthetic Insertion of Metal Nanoparticles and Their Cheaper Transition Metal Analogues within Mesoporous Matrix for Excitable Technical Applications

Porous silica nanoparticles (NPs)/mesoporous silica have been another important materials for a variety of applications, especially due to (1) their large surface area and pore volume for drug adsorption and loading within the pore channels; (2) the options for tuning adjustable pore size to control drug loading and drug release kinetics; (3) controlled and targeted drug delivery; (4) in vivo biosafety, biodegradation, biodistribution and excretion; (5) combinations with magnetic and/or luminescent compounds, which are potentially useful for bioimaging; and (6) the synthetic incorporation of metal nanoparticles and their multimetallic analogues for a variety of excitable applications, including as assets for carbon-free energy devices, options for achieving tunable catalytic activity, and as bioactive materials for bone regeneration. These mesoporous silica are originally derived from tri/tetra-alkoxysilane and have been proven to be excellent nanomaterials for further innovations with functional trialkoxysilane-derived formulations before or after achieving a controlled mesoporous geometry [3]. Accordingly, we attempted to explore the role of functional trialkoxysilane in both the synthetic incorporation of metal nanoparticles and their multimetallic analogues of cheaper transition metals, which not only act as a support matrix as enzyme-mimetic catalysts in biosensor applications but also serve as a vehicle in drug delivery.

One way of making mesoporous silica is to explore the sol–gel processing of alkoxysilane in the presence of a surface active material as a template for introducing pores in the silicate matrix, which can be explored again in the synthetic introduction of metal nanoparticles via organotrialkoxysilane-mediated reduction of metal cations where the silicate matrix is self-assembled, justifying the introduction of an organized mesoporous geometry in a variety of catalytic application, especially in green hydrogen production via catalytic decomposition of hydrogen-rich materials, such as hydrazine, and toxic dye degradation or water-splitting reaction [3]. These findings demonstrated the reduction of cheaper transition metal cations through NaBH_4_ in a controlled ratio of a noble metal, such as palladium, via reactive trialkoxysilane. We further innovated the process of making organic functionalized derivatives of cheaper transition metal catalysts, such as cobalt and nickel, with chelating agents, such as nitrilotriacetic acid (NTA) and phytic acid, which are susceptible to subsequent reduction under hydrothermal conditions [3] in nanostructured silicate domains as stable cheaper transition metal nanoparticles and are potentially viable for green hydrogen production. The tuning of catalytic activity with a small amount of a noble metal, such as palladium, may drastically alter the catalytic activity of such bimetallic nanocatalysts [3], which justifies the significant decrease in overvoltage associated with the water-splitting reaction in green hydrogen production. This process may be further innovated via the reduction of NTA-functionalized cobalt or nickel in the presence of organotrialkoxysilane-stabilized palladium nanoclusters, which allows the introduction of a porous bimetallic nanocatalyst upon calcination [3]. There is the further option for introducing trimetallic nanoparticles with the Pd-Co-Ni composition in the porous silicate matrix, resulting in the potent catalytic activity in green hydrogen production via the water-splitting reaction or the decomposition of hydrogen-rich stable organic chemical [3].

## 7. Reactive Organotrialkoxysilane-Derived Fluorescent Nanoparticles

The technology based on fluorescence exploration, either by using a known fluorophore or by introducing fluorescent nanoparticles, has received great attention [3]. Metal nanoparticles, especially gold nanoparticles, have shown fluorescence activity as a function of surface functionalization, and subsequent reactive organotrialkoxysilane-functionalized gold nanoparticles display excellent fluorescence activity [11,12,13,14,15,16,17,18,19,20,21,22,23,24,25,26,27,28,29]. Reactive organotrialkoxysilane protects the nanogeometry of noble metal nanoparticles and may also undergo faster electromeric interaction with a known fluorescent probe by acting as a spacer between the fluorescent probe and metal nanoparticles; this has been demonstrated in the decomposition and fluorescence sensing of hydrazine [3]. Reactive organotrialkoxysilane-derived gold nanoparticles have been found to display efficient fluorescence emission, and the emission intensity is found to be a function of 3-APTMS/3-GPTMA ratio, which ultimately controls the size of gold nanoparticles [3] and has been explored in sensitive dopamine sensing in cerebrospinal fluid [3]. The nature of the emission spectra is also found to be dependent on the organotrialkoxysilane-mediated synthetic strategy being either under microwave exposure or under an ambient condition; however, both types of gold nanoparticles are fluorescent. These findings have opened a new route of sensing and catalytic application in dealing with many unresolved scientific issues. 

## 8. Toxicities of Organotrialkoxysilane-Derived Functional Nanomaterials

The functionalities linked to trialkoxysilane as discussed in this article have already been proven to be biocompatible materials for deriving thin films that retain their biological activity in technological designs. Further amine group linked to trialkoxysilane has been found to be analogous to that of proteins in terms of reactivity and has been demonstrated in the selective interaction of biological components, especially when organotrialkoxysilane-derived silver nanoparticles are explored in antibacterial/antiviral action, justifying the selective conformation changes during antibacterial/antiviral action of these derived nanomaterials and further opening up a variety of ways for innovations with these potential technologies.
